# Effects of Magnolol and Honokiol on Adhesion, Yeast-Hyphal Transition, and Formation of Biofilm by *Candida albicans*

**DOI:** 10.1371/journal.pone.0117695

**Published:** 2015-02-24

**Authors:** Lingmei Sun, Kai Liao, Dayong Wang

**Affiliations:** 1 Department of Pharmacology, Medical School of Southeast University, Nanjing, China; 2 Department of Pathology and Pathophysiology, Medical School of Southeast University, Nanjing, China; 3 Key Laboratory of Developmental Genes and Human Disease in Ministry of Education, Medical School of Southeast University, Nanjing, China; Yonsei University, KOREA, REPUBLIC OF

## Abstract

**Background:**

The first step in infection by *Candida albicans* is adhesion to host cells or implanted medical devices and this followed by hyphal growth and biofilm formation. Yeast-to-hyphal transition has long been identified as a key factor in fungal virulence. Following biofilm formation, *C*. *albicans* is usually less sensitive or insensitive to antifungals. Therefore, development of new antifungals with inhibitory action on adhesion, yeast-hyphal transition and biofilm formation by *C*. *albicans* is very necessary.

**Methods:**

The effects of magnolol and honokiol on hypha growth were investigated using different induction media. Their inhibitory effects were determined using the 2,3-bis(2-methoxy-4-nitro-5-sulfophenyl)-2H-tetrazolium-5- carboxanilide assay, and biofilm thickness and viability were observed by a confocal scanning laser microscope. Mammalian cells were used in adhesion assays. Genes related to hyphae development and cell adhesions were analyzed by real-time reverse transcription-polymerase chain reaction. The exogenous cyclic adenosine monophosphate was used to determine the mechanisms of action of magnolol and honokiol. *Caenorhabditis elegans* was used as an *in vivo* model to estimate the antifungal activities of magnolol and honokiol.

**Results and conclusions:**

Magnolol and honokiol inhibited adhesion, the transition from yeast to hypha, and biofilm formation by *C*. *albicans* through the Ras1-cAMP-Efg1 pathway. Moreover, magnolol and honokiol prolonged the survival of nematodes infected by *C*. *albicans*. Magnolol and honokiol have potential inhibitory effects against biofilm formation by *C*. *albicans*.

**General Significance:**

This study provides useful information towards the development of new strategies to reduce the incidence of *C*. *albicans* biofilm-associated infection.

## Introduction

*Candida albicans* is one of the most common pathogens, and is associated with the occurrence of mortality rates as high as 35–50% [1,2]. The estimated annual cost of treating *Candida* infection exceeds $1 billion [1,2]. Infection by *C*. *albicans* involves adhesion to host cells or implanted medical devices as the first step, and this is followed by hyphal growth and biofilm formation [3–7]. The pathogenicity of *C*. *albicans* is closely related to its ability to change between two morphological forms, yeast and hyphae [7,8]. This transition is highly regulated and involves multiple interconnected signaling pathways, including the cyclic AMP-dependent Protein (cAMP) Kinase A and the Cph1-mediated Mitogen-Activated Protein Kinase (MAPK) [7]. *C*. *albicans* biofilm formation is very complex and the developmental processes involves the adhesion of yeast cells to the substrate, with the subsequent cell proliferation, elevation of hyphal growth, and production of extracellular matrix materials [8–10].^.^ The matured cells finally disperse from the biofilm into the surrounding environment [8]. Biofilm formation is an important step in pathogenesis because there after, *C*. *albicans* is usually less sensitive or insensitive to antifungals [11]. Therefore, development of new antifungals with inhibit action on adhesion, yeast-hyphal transition and biofilm formation of *C*. *albicans* would be a crucial strategy.

Natural products play an important role in drug discovery and development [12–14]. In the area of infectious disease, over 75% of drugs used in therapy are of natural origin [15]. *Magnolia officinalis* is a major component of herbal formulations such as Banxia-Houpu decoction and Huoxiang Zhengqi liquid which are used as remedies for illnesses like depression, coughing, asthma, liver disease, shoulder pain, urinary problems and diarrhea. Magnolol and honokiol, two neolignan compounds, are primarily isolated from the dried stem, root, or branch bark of *M*. *officinalis*. Previous studies have demonstrated that magnolol and honokiol have various pharmacological activities including antifungal [16–18]. However, the role of magnolol and honokiol in the inhibition of adhesion, transition from yeast to hypha, and biofilm formation by *C*. *albicans* as well the underlying mechanisms involved are still largely unclear.

In the present study, we first evaluated the inhibitory action of magnolol and honokiol against adhesion, yeast-hyphal transition and biofilm formation by *C*. *albicans in vitro*. The mechanisms of inhibition of biofilm formation by magnolol and honokiol were associated with the Ras1-cAMP-Efg1 pathway. Finally, the activity of magnolol and honokiol were investigated *in vivo*, using a nematode infection model. We believe our findings will be helpful in the future development of magnolol and honokiol as drugs against the formation of biofilm by *C*. *albicans*.

## Materials and Methods

### Strains and culture

The *C*. *albicans* strains used were SC5314 [19], CA1, CA2, CA3, CA4, CA10, CA127, CA129, CA132, CA135, and CA137 [20]. *C*. *albicans* CaSA1 [21] (*ura3*:: *imm434/ ura3*:: *imm434; CDR1-GFP-URA3*), a gift from Martin Schmidt (Des Moines University, Des Moines, USA), was used for cell adhesion assay. *C*. *albicans* YEM30 [22] was a gift from Michael D. Lafleur (Department of Biology, Northeastern University, USA). All strains were stored as frozen stocks of isolates in the culture medium (yeast extract–peptone–dextrose, YPD) supplemented with 10% (vol/vol) of glycerol at -80°C, and were subcultured twice at 37°C before use. Wild-type *Caenorhabditis elegans* strain N2 was grown at 20°C on nematode growth medium (NGM), spotted with *Escherichia coli* OP50. *E*. *coli* OP50 was grown overnight in Luria broth at 37°C.

### Chemicals

**Fig 1:**
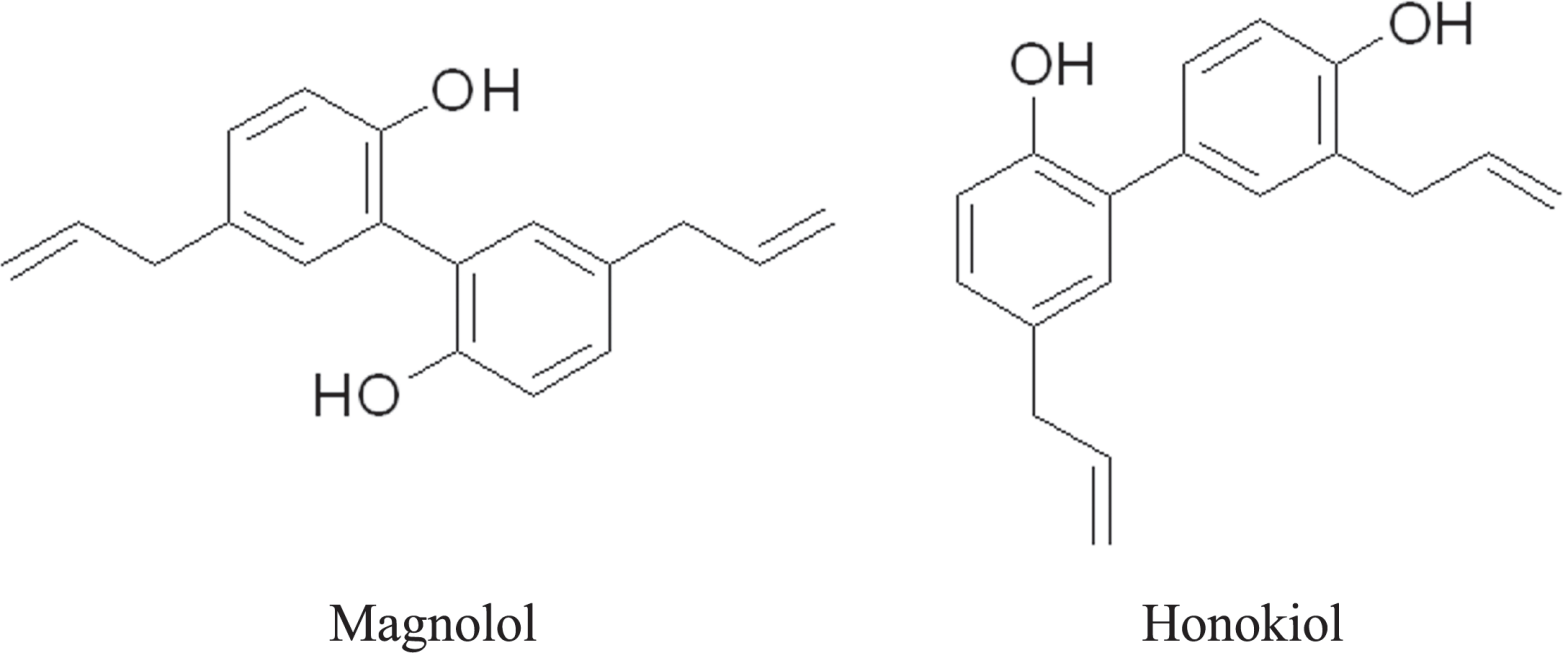
Structures of honokiol and magnolol.

Magnolol (5,5’-diallyl-2,2’-dihydroxybiphenyl) and honokiol (5,5’-diallyl-2,4’-dihydroxybiphenyl) (Fig. 1) were obtained from Xi'an Yuquan Biological Technology Co., Ltd. Amphotericin B was purchased from Sigma-Aldrich. Stock solutions were prepared in dimethyl sulfoxide at 10240 μg/mL. 2,3-bis(2-methoxy-4-nitro-5-sulfophenyl)-2H-tetrazolium-5- carboxanilide (XTT) and menadione were purchased from Sigma Chemical

### Antifungal activities of compounds

The antifungal activities of all tested compounds were tested by the broth microdilution method according to CLSI standard M27-A3 [23] with a final inoculum of 0.5 × 10^3^ to 2.5 × 10^3^ cells/mL. The test was carried out in RPMI 1640 medium adjusted to pH 7.0 with 0.165 M morpholinepropanesulfonic acid [MOPS] buffer in 96-well flat-bottomed microtitration plates. After incubation at 37°C for 48 h, minimum inhibitory concentrations (MICs) were determined by measuring the optical density at 600 nm with the BioTek Synergy 4 microplate reader (BioTek Instruments Inc., USA). The background optical densities were subtracted from that of each well. The MICs were defined as the concentrations of drug that reduced growth by 80% compared to that of organisms grown in the absence of drug. The experiments were performed in triplicate.

### Measurement of minimum fungicidal concentrations (MFCs)

MFCs were determined as described previously [24]. After the MIC was determined, a 5-μL aliquot was taken from each well and added to 200μL of compound-free fresh YPD medium. After 48 h of incubation, the MFC was determined as the lowest concentration of the test compound in which no recovery of microorganisms was observed.

### Effect of magnolol and honokiol on *C*. *albicans* growth

*C*. *albicans* SC5314 with different starting inoculum of 10^6^, 10^7^, 10^8^ cells/mL in YPD medium were exposed at different concentrations of magnolol and honokiol. Samples were taken at 4, 8, 12, and 24 h and yeast counts were determined using 10-fold dilutions plated on YPD plates which were incubated at 37°C for 48 h. Using a 20-μL sampling volume, the lower limit of accurate and reproducible quantitation was 50 CFU/mL for each of the isolates. All experiments were performed in triplicate and repeated on two different days. A fungicidal effect was defined as a reduction in ≥3 log_10_ CFU/mL compared to initial inoculum [25].

### Filamentation assay—solid spider media for colony morphology assay

Spider media agar plates with or without various concentrations of compounds were used in this study. SC5314 cells pregrown overnight in YPD media were diluted into spider liquid media to a final concentration of 1000 cells/mL. And then 50 μL of the solution were spread onto the plates. Plates were incubated at 37°C for 3 days. Images of colony edges were obtained using a microscope (Olympus IX71, Olympus Co., Tokyo, Japan). The experiments were performed in triplicate.

### Filamentation assay—RPMI 1640, GlcNAc, or 10% FBS YPD liquid medium

*C*. *albicans* SC5314 were grown overnight in YPD medium. And then, 1 × 10^6^ cells/mL with or without compounds were incubated in RPMI 1640 medium, GlcNAc (0.5% GlcNAc, 0.5% peptone, 0.3% KH_2_PO_4_) or 10% FBS YPD medium at 37°C for 4 h. Inhibition quantification of the yeast-to-hyphal-form transition was accomplished by counting the number of individual budded cells *versus* the number of hyphae in the population as previously described [26]. More than 100 cells were counted for each well in duplicate, and all assays were repeated for five times. Images of cells were obtained using a microscope. The experiments were performed in triplicate.

### Biofilm formation—before the biofilm adhesion phase

Various concentrations of compounds ranging from 8 to 64 μg/mL were prepared in RPMI 1640 medium in 96 well plates (Costar, Corning Inc., USA). Wells without test compounds served as controls. Amphotericin B (8 to 64 μg/mL) was used as a standard antifungal agent. Cell suspensions of 1 × 10^6^ cells/mL were prepared in RPMI 1640 medium. And then 100 μL of solution was inoculated into 96-well polystyrene plates. After incubation at 37°C for 48 h, nonadhered cells were removed by sterile PBS, and biofilm growth was analyzed with XTT assay, as described [20]. The absorbance of wells was measured with the BioTek Synergy 4 microplate reader at 490 nm. The experiments were performed in triplicate.

### Biofilm formation—after the biofilm adhesion phase

Cell suspensions of 1 × 10^6^ cells/mL were prepared in RPMI 1640 medium, and 100 μL of cell suspensions was inoculated into 96-well polystyrene plates to incubate at 37°C for 6 h or 24 h to allow attachment of cells to the solid surface. Nonadhered cells were removed by sterile PBS. And then 100 μL of various concentrations of compounds in RPMI 1640 medium were added to each well. The plates were incubated at 37°C for 48 h to allow for biofilm formation. Biofilm growth was analyzed with XTT assay as described above. The experiments were performed in triplicate.

### Analysis of *C*. *albicans* biofilm formation using confocal scanning laser microscope (CLSM)

The *C*. *albicans* strain SC5314 (1 × 10^5^ cells/mL) was cultured in RPMI 1640 medium at 37°C for 48 h with or without tested compounds. After incubation, the supernatant was aspirated, and nonadherent cells were removed by washing with sterile PBS. The biofilm were co-stained with 10 μg/mL of fluorescein diacetate (FDA) and 5 μg/mL of propidium iodide (PI) for 30 min. The images were taken by confocal laser-scanning microscope (Olympus Fluoview FV1000). Laser beams with 488 and 555-nm excitation wavelengths were used for FDA and PI imaging, respectively. A detailed three-dimensional image of biofilm was performed using Z-stacks (depending on the height of the biofilm). Cell viability was also able to be assessed since the healthy cells would hydrolyze FDA so as to accumulate the green fluorescence, whereas the dead cells were stained as red due to the PI labeling. Red or green fluorescence intensity was calculated using histogram in ImageJ.

### *In vitro* biofilm growth on titanium sheet

Titanium is widely employed for implant manufacturing due to its good biocompatibility and mechanical properties, but infection remains a cause of failure leading to removal. The titanium surface is not antimicrobial by itself, so it could be used as support for a *Candida* biofilm in this investigation [27]. The effect of honokiol or magnolol on biofilm growth on titanium sheet was examined as described previous [28]. After autoclaved, the squares (1.0 × 1.0 cm) were transferred to 35 mm glass bottom dishes (NEST Biotech, China) and incubated with 1 × 10^5^ cells/mL SC5314 in RPMI 1640 medium at 37°C for 2 h. The squares were washes with PBS and transferred to new dishes with or without tested compounds. After incubation at 37°C for 48 h, the dishes were photographed, and images of titanium sheet edges were visualized under a microscope. The bound biofilm was measured after drying of titanium sheet in a chemical hood. The experiments were performed in triplicate.

### Cell adhesion assay

An immortalized rat hepatic stellate cell line HSC-T6 was used in this study [29]. HSC-T6 cells were maintained in DMEM supplemented with 10% heat-inactivated fetal bovine serum at 37°C in a humidified atmosphere of 5% CO_2_. HSC-T6 cells were grown to confluence on 48 well plates. Media were decanted, and the plates were carefully washed three times with PBS to remove unbound cells. And then 500 μL of 1 × 10^7^ cells/mL *C*. *albicans* CaSA1 cells mixed with various concentrations of compounds were plated into each well. After incubation at 37°C for 2 h, the media were decanted, and the monolayers were washed carefully three times with PBS. The number of adherent *C*. *albicans* cells was determined by scraping the bottom of the 48-well plate with a sterile scraper. Five-fold serial dilutions were plated on YPD agar to determine viability. Also, both DIC and fluorescence images were acquired using a fluorescence microscope (Olympus IX71, Olympus Co., Tokyo, Japan). The experiments were performed in triplicate.

### Quantification analysis by real-time reverse transcription-polymerase chain reaction (RT-PCR)

The total RNAs were isolated using the hot phenol method as previously described [30]. *C*. *albicnas* was grown overnight in YPD medium and diluted to a cell density of 1.0 × 10^6^. Yeasts were incubated at 37°C for 12 h followed by centrifugation at 4°C. Approximately 1 μg of total RNA was used to synthesize cDNA using random primers and AMV reverse transcriptase (Promega, WI, USA). Gene expression levels of *GSP1*, *RAS1*, *EFG1*, *TEC1*, *CDC35*, *ALS3*, *HWP1* and *ECE1* were analyzed by the real-time RT-PCR. Primer sequences used for amplification of specific genes are shown in S1 Table. Real-time RT-PCR was performed in 8-tube strips in triplicate. Each reaction containing 1 × GoTaq qPCR Master mix (Promega, Madison, WI), 0.2 μM forward primer and reverse primer, and 1 μL template cDNA in a final volume of 20 μL. *GSP1*, which was not transcriptionally regulated in the morphogenesis switch, served as the internal control. Cycling profile included 35 cycles of 95°C for 60 sec, 60°C for 60 sec and 72°C for 45 sec. Data acquisition and the analysis of the RT-PCR assay were performed using an ABI 7500 Real-Time PCR System (Applied Biosystems). The transcript level of detected genes was calculated using the formula 2^-ΔΔ*CT*^.

### cAMP Rescue Experiments

Grown overnight *C*. *albicans* SC5314 cells were diluted to 2×10^5^ cells/mL with RPMI1640 medium. Dibutyryl-cAMP (Sigma) was added to the cultures with a final concentration of 5 mM immediately following the addition of 8 μg/mL magnolol or honokiol. The free-drug treatment cells with or without dibutyryl-cAMP (dbc-AMP) served as control. After 4 h incubation, cells were visualized by an Olympus microscope.

### Toxicity assessment of compounds in HSC-T6 cells

The HSC-T6 cells were seeds into 96-well plates at 1 × 10^4^ cells/mL and grown at 37°C overnight. The media was removed and replaced with fresh media containing compounds followed by incubation for further 24 h. The CCK-8 assay was used to evaluate cell viability. The cells were washed with D-Hank’s buffer, and 200 μl of WST-8 solution (Dojindo Molecular Technologies, Inc., USA) was introduced to each well to incubate for an additional 3 h at 37°C. The optical density (OD) of each well at 450 nm was examined with the BioTek Synergy 4 microplate reader (BioTek Instruments Inc., USA). The experiments were performed in triplicate.

### Antifungal activity and evaluation of fugal burden in a nematode model of infection

*C*. *elegans* survival analysis was performed as previously described [31]. Age synchronous populations of L4-larvae were prepared, and exposed to *C*. *albicans* SC5314. For the pathogen assay, 75 mg/mL of fluoro-29-deoxyuridine (FUdR) was added to the assay plates to prevent the growth of progeny. *C*. *albicans* was seeded on killing plates containing modified NGM (0.35% instead of 0.25% peptone). *C*. *albicans* was incubated first for 24 h at 37°C and then for 24 h at 25°C. Young adult animals were exposed to the *C*. *albicans* lawns for 2 h and washed three times with sterile M9 buffer, then moved to NGM in the presence or absence of antifungal agents. The infection was started by adding 60 animals to each plate at 25°C, and scored for dead or live every 12 h. Animals were scored as dead if no response was detected after prodding with a platinum wire. Three replicates were analyzed for each experiment.

The number of *C*. *albicans* colony-forming Units (CFU) in *C*. *elegans* was quantified based on the protocol described previously [32]. *C*. *albicans* lawns were prepared on BHI as described above [32]. Young adult animals were exposed to the lawns for 2 h and then moved to liquid media (80% M9 buffer, 20% BHI, and 90 μg/mL kanamycin) and incubated at 20°C in the presence or absence of antifungal agents. After 24 h incubation, three groups of twenty worms each were washed three times with 1 mL of M9 medium to remove surface *Candida*. Each group of twenty worms was then disrupted using a homogenizer and plated on YPD agar containing kanamycin (45 μg/mL), ampicillin (100 μg/mL), and streptomycin (100 μg/mL). The plates were incubated for 48 h at 37°C and colonies were counted to determine CFU per nematode. Three replicates were analyzed for each experiment.

### Statistical analysis

All data were presented as means ± standard error of the mean (S.E.M.). Graphs were generated using Microsoft Excel (Microsoft Corp., Redmond, WA). Statistical analysis was performed using SPSS 12.0 (SPSS Inc., Chicage, USA). Differences between groups were determined using analysis of variance (ANOVA). A P-value<0.05 was defined as statistically significant. The lifespan data were statistically analyzed using a 2-tailed 2 sample t-test (Minitab Ltd., Coventry, UK).

## Results

### Inhibitory activity of magnolol and honokiol against planktonic growth of *C*. *albicans*

Magnolol and honokiol exhibited similar and potent inhibitory activitiy against clinically isolated *C*. *albicans* strains (SC5314, CA1, CA2, CA3, CA4, CA10, CA127, CA129, CA132, CA135, and CA137), with MICs in the range of 16–32 μg/mL (S2 Table). In all cases the MIC was equivalent to the MFC. After 48 h of exposure to treatments, samples were re-inoculated in YPD medium. After an additional 48 h incubation, no growth was detected in cultures exposed to the MIC values of magnolol and honokiol with the MIC values.

**Fig 2:**
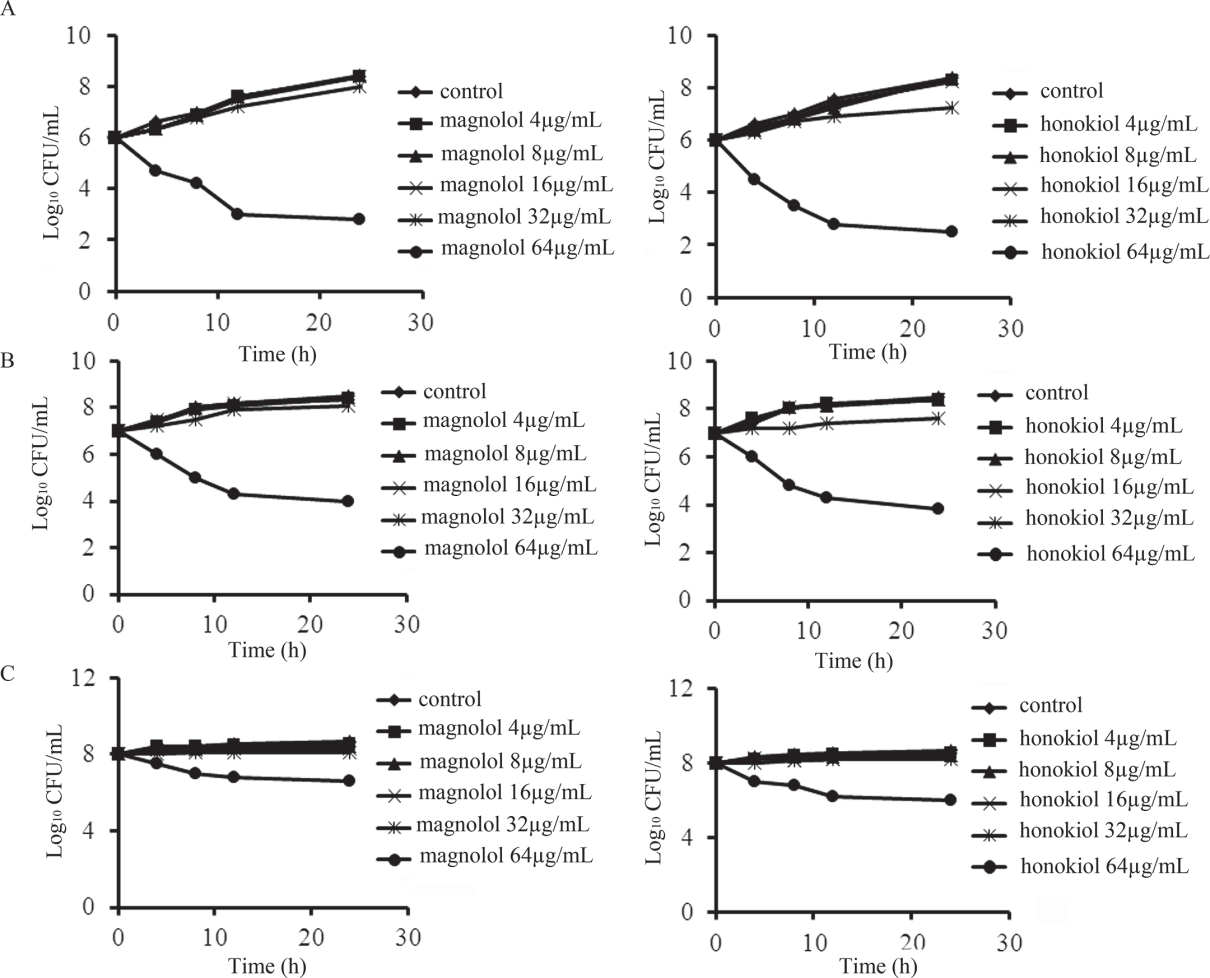
Time-growth curves of different concentration of magnolol and honokiol on *C*. *albicans* SC5314 at different initial inoculum levels. The initial inoculums were 1×10^6^ cells/mL (A), 1×10^7^ cells/mL (B), and 1×10^8^ cells/mL (C). Aliquots were obtained at the indicated time points and serially dilutions were spreads on YPD agar plates. Colony counts were determined after 48 h incubation.

The effect of magnolol and honokiol on growth of *C*. *albicans* was further investigated. It was observed that the fungicidal activities of these compounds depended greatly on the initial inoculum (Fig. 2). Time-growth curves indicated that, compared with the control group, 4, 8, or 16 μg/mL of compounds could not affect the growth of *C*. *albicans* after 24 h of incubation. But 32 μg/mL of magnolol and honokiol slightly slowed down the growth of *C*. *albicans* when incubated with initial inoculum of 1.0×10^6^ or 1.0×10^7^ cells/mL (Fig. 2A and 2B). A fungicidal effect was observed when 64 μg/mL of magnolol or honokiol was used with initial inoculum of 1.0×10^6^ or 1.0×10^7^ cells/mL. When the initial inoculum of 1.0×10^8^ cells /mL was used, only 64μg/mL of magnolol or honokiol had a reduction in the growth of *C*. *albicans*, and no fungicidal activity was observed (Fig. 2C). Although the chemical structures of magnolol and honokiol are different, particularly in the position of the hydroxyl group on the aromatic ring, this difference appears not to influence their biological activity on *C*. *albicans*.

### Inhibitory activity of magnolol and honokiol against the transition of *C*. *albicans* from yeast to hyphae

**Fig 3:**
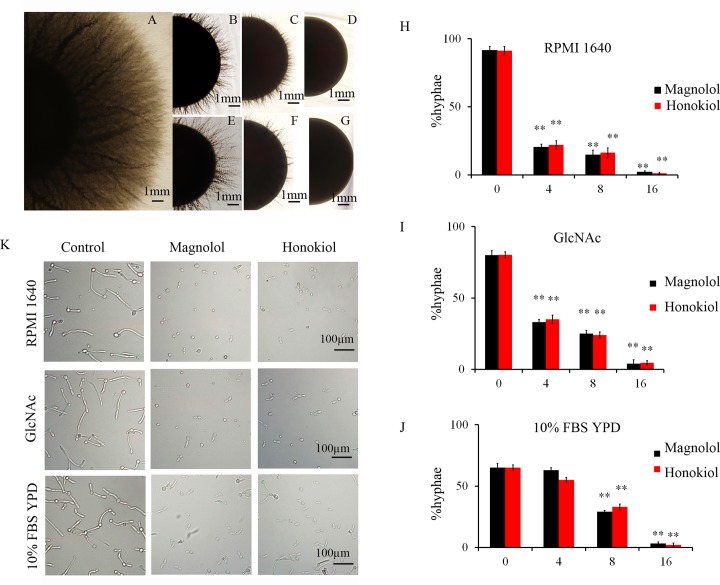
Inhibition of *C*. *albicans* filamentation by honokiol and magnolol in different hyphal-inducing media. (A-G) Colonies were grown on solid spider media agar plates overlaid with vehicle (control), magnolol, or honokiol. Images of colony edges were obtained using a microscope. (A) Solvent controls; (B) 4 μg/mL of honokiol; (C) 8 μg/mL of honokiol; (D) 16 μg/mL of honokiol; (E) 4 μg/mL of magnolol; (F) 8 μg/mL of magnolol; (G) 16 μg/mL of magnolol. (H-J) Honokiol and magnolol inhibited *C*. *albicans* filamentation induced on RPMI1640, GlcNAc, or FBS YPD medium. *C*. *albicans* CASA1 (1 × 10^6^ cells/mL) with honokiol, or magnolol was incubated in (H) RPMI1640, (I) GlcNAc, or (J) 10% FBS YPD medium at 37°C for 4 h. Bars represent means ± S.E.M. **P < 0.01. (K) representative images of *C*. *albicans* cells obtained using a fluorescent microscope. Honokiol and magnolol were added at a concentration of 16 μg/mL.

The transition from yeast to hyphae is an important virulence-mediating attribute of *C*. *albicans*. Formation of hyphae helps *C*. *albicans* in penetrating the host tissues with subsequent invasive growth that leads to the establishment of systemic infection. Conversion of yeast cells to filamentous forms is critical for biofilm biogenesis, which provides strength and support to the developing heterogeneous biofilm structure. In the present study, we observed that magnolol and honokiol at a low concentration of 4 μg/mL effectively inhibit transition from yeast to hyphae on the solid spider agar plates (Fig. 3A-3G) and at 16 μg/mL both compounds completely blocked this transition (Fig. 3D and 3G).

Hyphal morphogenesis can be induced by multiple stimuli [7]. In addition to the nutrient-poor spider medium, liquid RPMI 1640, GlcNAc and mammalian serum can also induce the formation of hyphae [7]. We further discovered that both magnolol and honokiol at a low concentration of 4μg/mL significantly inhibited the hyphae formation in RPMI 1640 or GlcNAc media (Fig. 3H-3I, and 3K). In contrast, 4 μg/mL of magnolol or honokiol did not obviously influence the hyphae formation in 10% FBS YPD medium (Fig. 3J-3K). Magnolol and honokiol at different concentrations had similar effects on hyphae growth in different hyphae-inducing media (Fig. 3H-3K). Therefore, our data suggest that both magnolol and honokiol have the potential to inhibit the transition of *C*. *albicans* from yeast to hyphae.

### Inhibitory effects of magnolol and honokiol on biofilm growth at different stages in *C*. *albicans*

**Fig 4:**
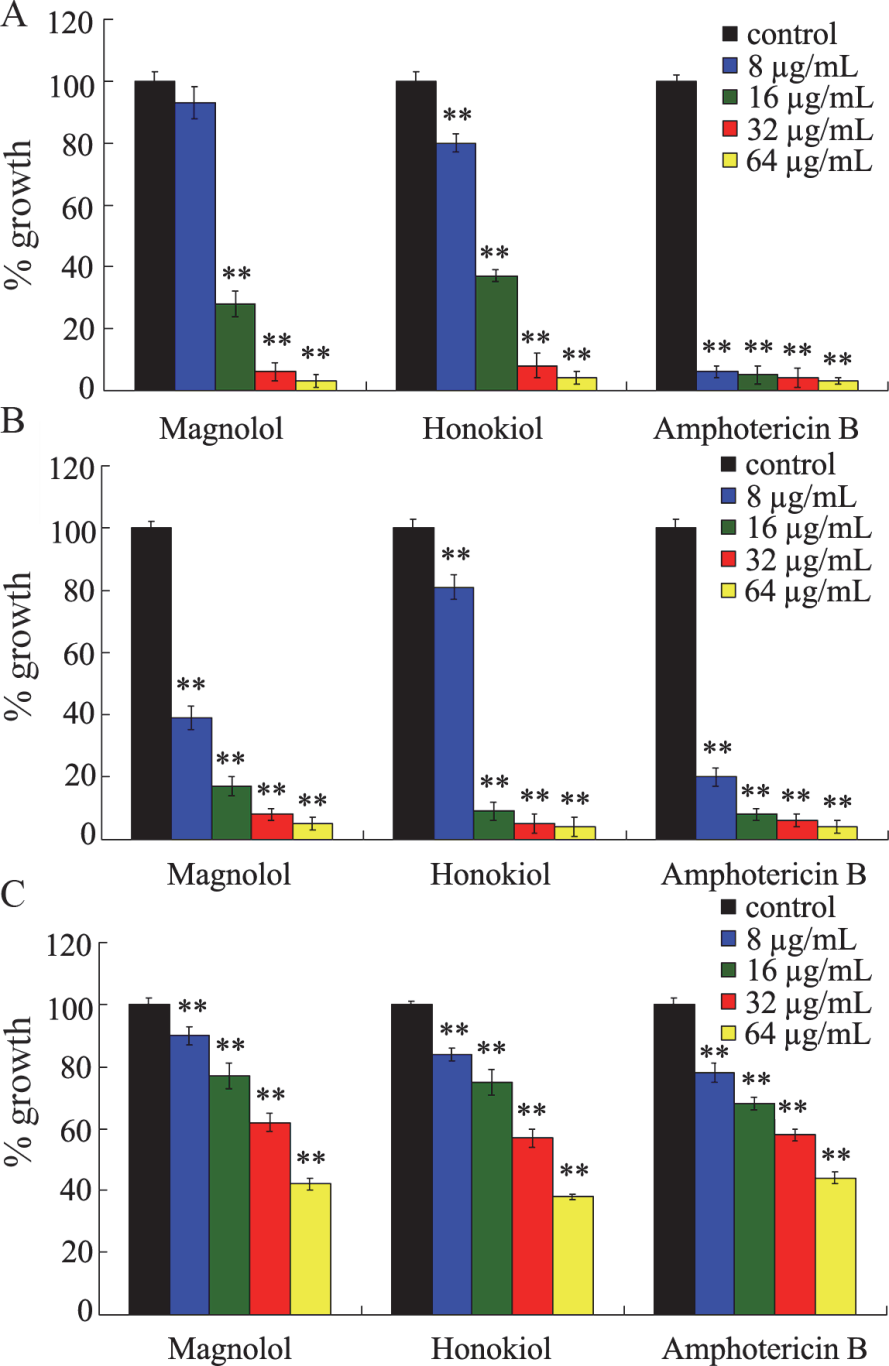
Inhibition of *C*. *albicans* biofilm growth by magnolol and honokiol. (A) *C*. *albicans* SC5314 cells were incubated continuously in the presence of compounds at 37°C for 48 h. (B) *C*. *albicans* SC5314 cells were allowed to adhere for 6 h then magnolol or honokiol was added and incubated further for 48 h at 37°C. (C) *C*. *albicans* SC5314 cells were allowed to adhere for 24 h then magnolol or honokiol was added and incubated further for 24 h at 37°C. Amphotericin B served as a positive control. Following incubations, the bound cells were detected with an XTT assay. Bars represent means ± S.E.M. **P < 0.01.

The development of *C*. *albicans* biofilm involves several specific stages including the early phase, the developmental phase, and the biofilm maturation stage. It was found that magnolol and honokiol inhibited biofilm formation in a dose-dependent manner (Fig. 4). During the early phase (that is immediately after adhesion), biofilm formation in the 16 μg/mL magnolol or honokiol group was less than 50% as compared with the control group (Fig. 4A). At the developmental phase, treatment with 16 μg/mL of magnolol and honokiol inhibited more than 80% biofilm formation by *C*. *albicans* (Fig. 4B). Furthermore, the inhibitory effects of 16–64 μg/mL magnolol or honokiol on biofilm formation were comparable to those of amphotericin B, a “gold standard” drug used in the treatment of *Candida* infections (Fig. 4B). At the biofilm maturation stage, treatment with 64 μg/mL of magnolol and honokiol decreased more than 50% biofilm formation, which was similar to the function of amphotericin B (Fig. 4C).

### The effects of magnolol and honokiol on biofilm thickness and viability

**Fig 5:**
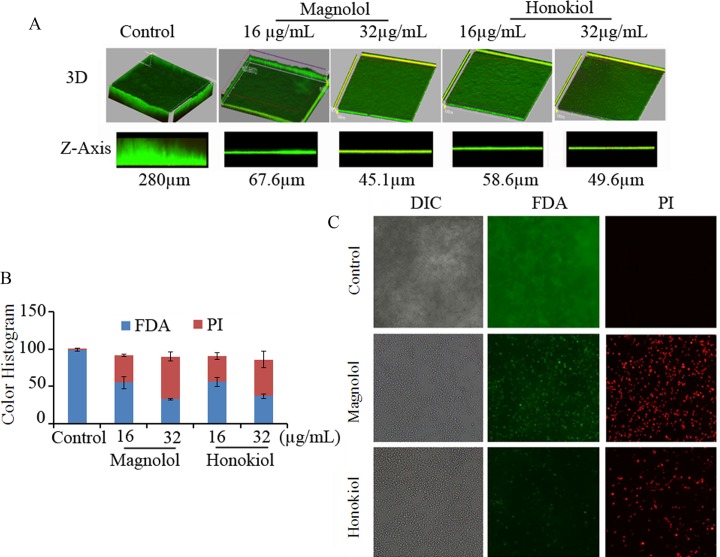
CLSM analysis showing the inhibition of biofilm development by honokiol and magnolol. (A) The 3D images showing the biofilm in *C*. *albicans* SC5314 treated with drugs. The rotated 3D-view and the side view Z-axis were showed. (B) Red or green fluorescence intensity was calculated using histogram in ImageJ. Bars represent means ± S.E.M. (C) Fluorescent microscopic was used to visualize formation of biofilm stained with FDA and PI in *C*. *albicans* SC5314 treated with 16 μg/mL magnolol or honokiol.

The anti-biofilm effects of magnolol and honokiol were further confirmed by CLSM analysis. To analyze the effects of magnolol and honokiol on biofilm thickness, three dimensions (3D) views were taken for each sample and Z-stacks were prepared to compare the thickness. In the control experiment, *C*. *albicans* cells formed a thick biofilm with an average Z-axis of 280 μm (Fig. 5A). In contrast, treatment with magnolol at concentrations of 16 and 32 μg/mL reduced the thickness of the biofilm to 58.6 μm and 49.6 μm, respectively (Fig. 5A). Similarly, treatment with honokiol at concentrations of 16 and 32 μg/mL decreased the thickness of biofilm to 67.6 μm and 45.1 μm, respectively (Fig. 5A).

Furthermore, both magnolol and honokiol at 16 μg/mL significantly decreased the viability of the biofilm as indicated by FDA labeling and increased the dead *C*. *albicans* cells as indicated by PI labeling (Fig. 5B-5C).

### Inhibition of biofilm formation on titanium sheet by magnolol and honokiol

**Fig 6:**
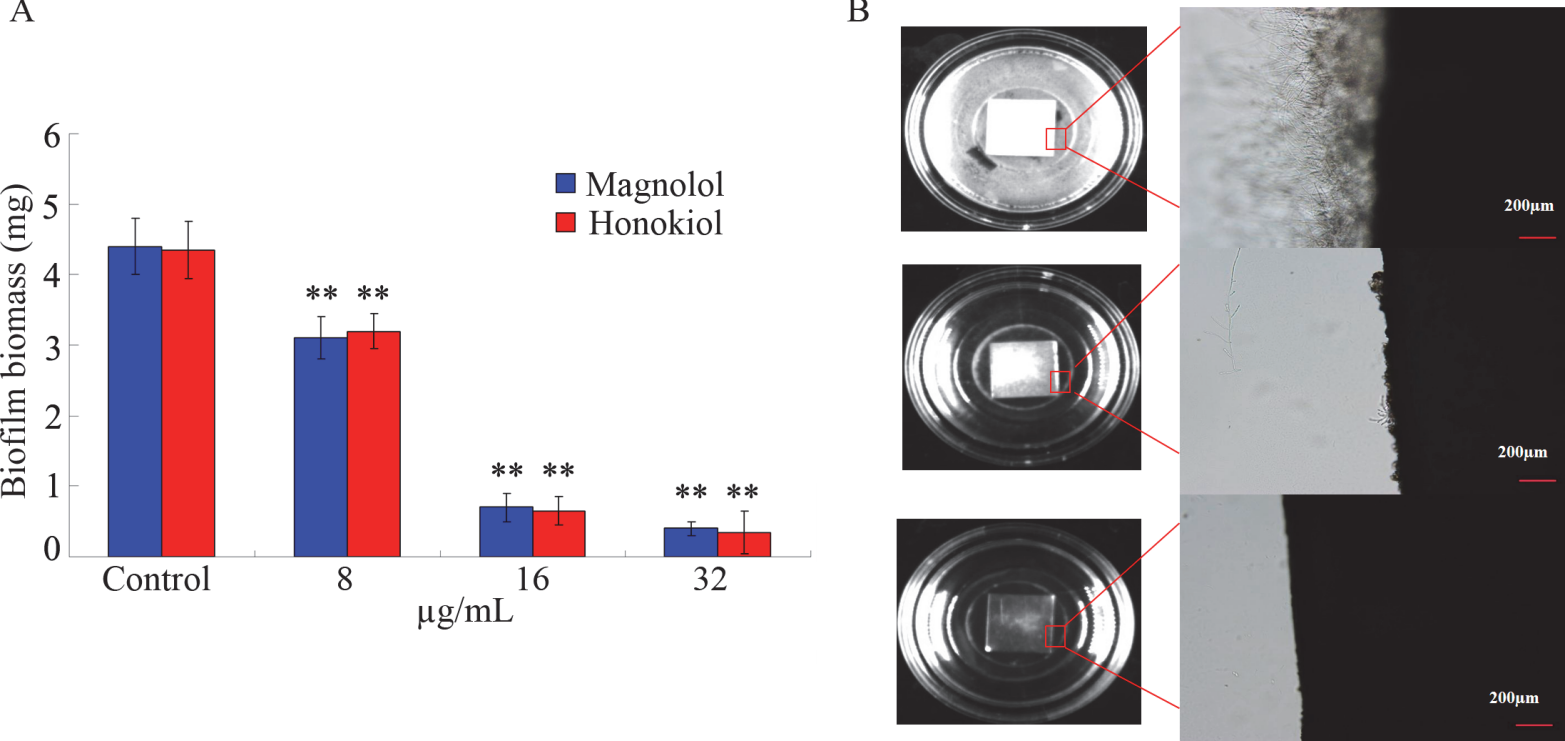
Magnolol and honokiol inhibited biofilm formation by *C*. *albicans* on titanium sheets. (A) Comparison of *C*. *albicans* biofilm biomass. Bars represent means ± S.E.M. **P < 0.01. (B) Biofilms of *C*. *albicans* SC5314 cells formed on titanium sheets photographed 48 h after treatment. The enlarged views were also showed. The treatment concentration for magnolol and honokiol was 16 g/mL.

Fungal biofilm formation on implanted medical devices is a serious medical problem, which can lead to life-threatening systemic infections [6,28]. We used the titanium sheet assay [27] to simulate the formation of biofilm by *C*. *albicans* on medical devices. We confirmed that *C*. *albicans* SC5314 cells efficiently formed biofilms on titanium sheet (Fig. 6). In contrast, magnolol and honokiol at concentrations of 8–32 μg/mL significantly suppressed biofilm formation (Fig. 6A and 6B). The edges of the titanium sheets treated with 16 μg/mL of magnolol or honokiol were clearer with less *C*. *albicans* growth than that of the vehicle control (Fig. 6B).

### Inhibition of *C*. *albicans* adhesion to the surface of HSC-T6 cells by magnolol and honokiol

**Fig 7:**
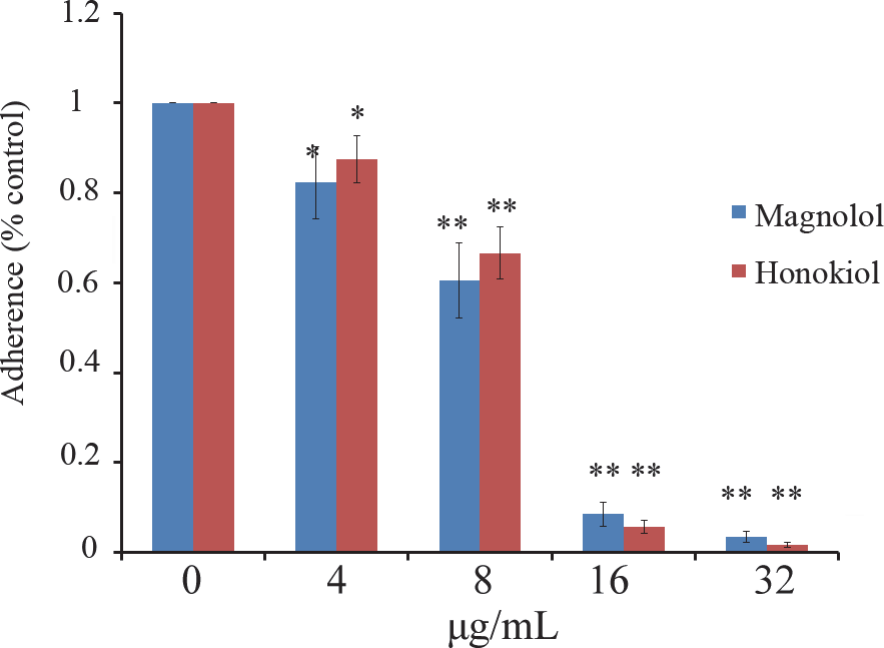
Magnolol and honokiol inhibited the adhesion of *C*. *albicans* CASA1 to HSC-T6 cells. Effects of magnolol or honokiol treatment on adhesion of *C*. *albicans* CASA1 cells to HSC-T6 cells. Bars represent means ± S.E.M. *P < 0.05, **P < 0.01.

In addition to the experiments on inert surfaces, we further investigated the effects of magnolol and honokiol treatment on *C*. *albicans* adherence to the surface of mammalian cells. Magnolol and honokiol at concentrations of 4–32 μg/mL significantly inhibited the adhesion of *C*. *albicans* cells to the surface of HSC-T6 cells (Fig. 7). Moreover, the fluorescent images also confirmed that treatment with 16 μg/mL of magnolol or honokiol significantly inhibited adhesion to HSC-T6 cells (S1 Fig.).

### Ras1-cAMP-Efg1 pathway is involved in the mechanisms of action of magnolol and honokiol

**Fig 8:**
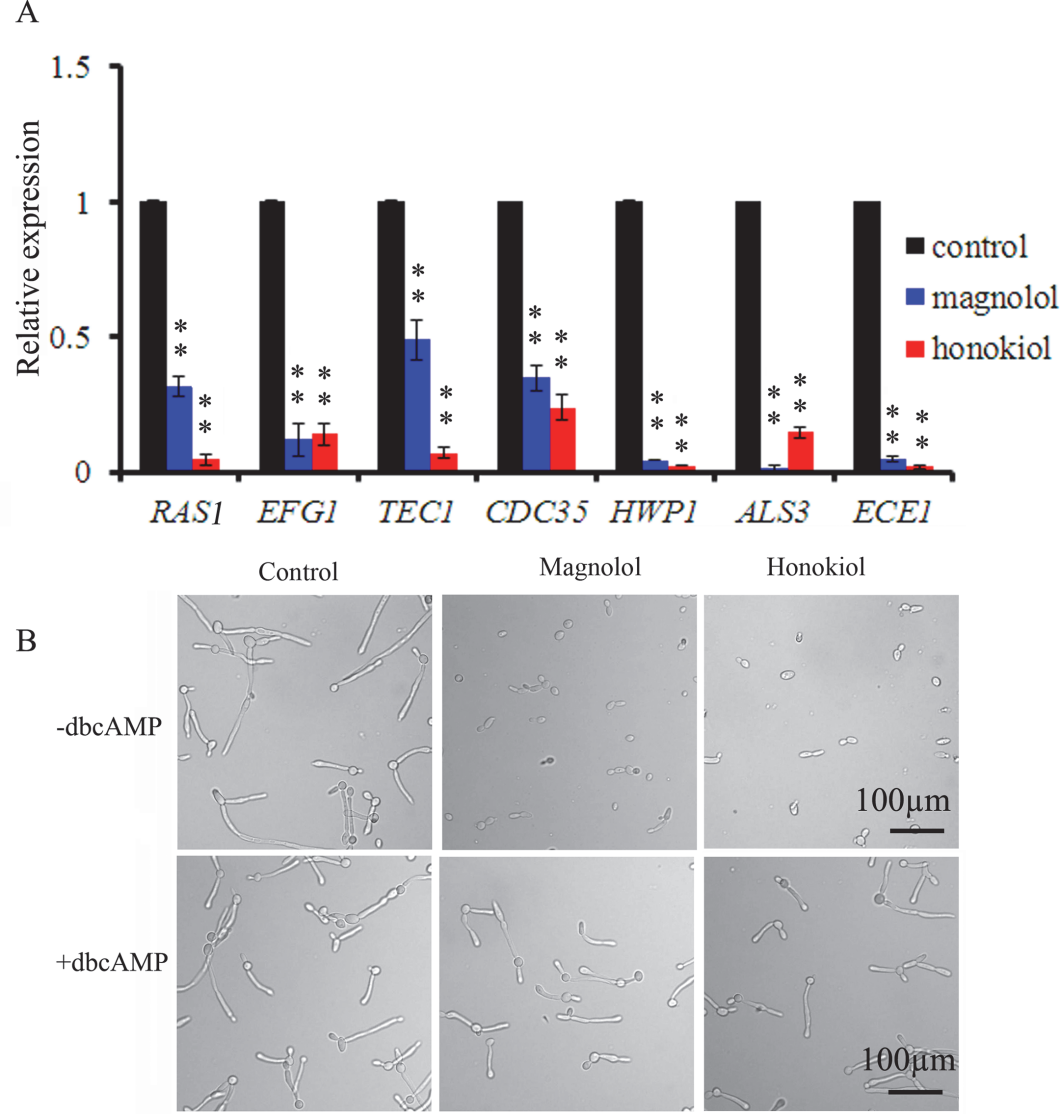
Magnolol or honokiol inhibited hyphal development induced by Ras1-cAMP-Efg1 pathway. (A) Expression patterns of genes encoding Ras1-cAMP-Efg1 pathway and cell adhesion related genes in *C*. *albicans* SC5314 was treated with 16 μg/mL of magnolol or honokiol for 12 h at 37°C. Gene expression was indicated as a fold change relative to that of the control group after real-time RT-PCR assay. Bars represent means ± S.E.M. **P < 0.01. (B) Exogenous cAMP restored the magnolol or honokiol-inhibited hyphae formation. SC5314 cells were grown in RPMI 1640 medium with indicated treatment. After 4 h, the morphology was visualized by microscopy.

To understand the molecular basis for magnolol and honokiol inhibiting hyphae growth and cell adhesion, we investigated the expression of genes related to hyphae growth and cell adhesion in *C*. *albicans* treated with both compounds. Four genes (*RAS1*, *EFG1*, *TEC1*, and *CDC35*) associated with the Ras1-cAMP-Efg1 pathway were examined. After treatment with 16 μg/mL of magnolol or honokiol, the expression levels of *RAS1*, *EFG1*, *TEC1*, and *CDC35* genes were significantly decreased (Fig. 8A). Ras1p is a GTPase that plays a role in inducing hyphal formation by activating both the Ras1-cAMP-Efg1 pathway and MAPK cascade pathway. *EFG1* is a transcription factor in the Ras1-cAMP-Efg1 pathway which plays an important role in regulating the expression of some hypha-specific genes, including *ECE1*, *HWP1 and ALS3*. In our study, following treatment with 16 μg/mL of magnolol or honokiol, the expression levels of all three genes examined including *ECE1*, *HWP1*, and *ALS3* were significantly decreased (Fig. 8A). Next, we investigated whether magnolol and honokiol can also down-regulate the expression levels of these genes in other *C*. *albians* strains. For this purpose, we used the strain YEM30, and the results in YEM30 were similar to those in SC5314 (S2 Fig.). Based on these results, we therefore speculated that the inhibition of the transition from yeast to hyphae by magnolol and honokiol may be related to down-regulation of the Ras1-cAMP-Efg1 pathway. To verify this hypothesis, we conducted the exogenous cAMP rescue experiments. The results revealed that exogenous cAMP restored the hyphal formation in the magnolol and honokiol treatment groups (Fig. 8B). Together, these data suggest that magnolol and honokiol may inhibit the filamentous growth through the Ras1-cAMP-Efg1 pathway.

### Magnolol and honokiol exhibited antifungal activity in the *in vivo C*. *elegans* assay system

**Fig 9:**
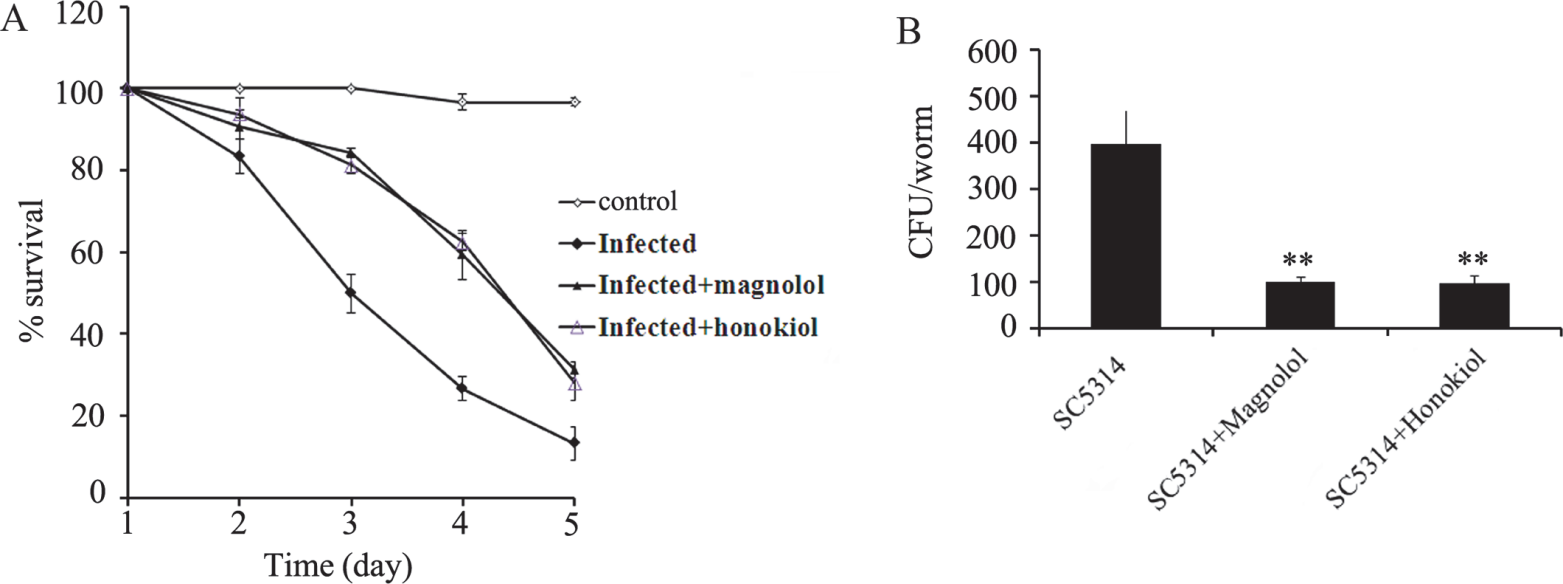
Antifungal activity assay for magnolol and honokiol in nematodes infected with *C*. *albicans* SC5314. (A) Survival curves of nematodes infected with *C*. *albicans* SC5314. (B) CFU of *C*. *albicans* SC5314 in nematodes. The concentration for magnolol or honokiol was 16 μg/mL. Control, uninfected. Bars represent means ± S.E.M. **P < 0.01.

In *C*. *elegans*, we found that infection with *C*. *albicans* SC5314 induced a significant (*P* < 0.0001) decrease in lifespan of the nematodes compared with control (Fig. 9A). Treatment with 16 μg/mL of magnolol or honokiol significantly (*P* < 0.0001) increased the lifespan of nematodes infected by *C*. *albicans* SC5314 (Fig. 9A). Moreover, we found that treatment with 16 μg/mL of magnolol or honokiol significantly reduced the CFU in the body of nematodes (Fig. 9B). Thus, magnolol and honokiol reduced the colonization of *C*. *albicans* in the body of nematodes and thereby increase the lifespan of infected nematodes.

### Toxicity assessment of magnolol and honokiol in HSC-T6 cells

Finally, we investigated the safety profile of magnolol and honokiol using mammalian HSC-T6 cells. We found that both magnolol and honokiol at concentrations of 4–32 μg/mL did not influence the viability of mammalian HSC-T6 cells (S3 Fig.) or affect the morphology, longevity, development, and reproduction of nematodes (data not shown). These data suggest that magnolol and honokiol at the concentrations tested may not have adverse effects on these organisms.

## Discussion

In the present study we investigated the effects of magnolol and honokiol, two neolignan compounds isolated from *M*. *officinalis*, on adhesion, yeast-hyphal transition, and biofilm formation by *C*. *albicans*. It has been previously reported that the MICs of both magnolol and honokiol were 25 μg/mL against *C*. *albicans* KCTC 1940 and *Epidermophyton floccosum* KCTC 1246, while for other strains such as *Trichophyton mentagrophytes* KCTC 6077, *Microsporium gypseum* KCTC 1252, and *Cryptococcus neoformans* KCTC 7224, the antifungal effect of honokiol was evaluated to be higher than that of magnolol [16]. Although the chemical structures between magnolol and honokiol differ in the position of the hydroxyl group on aromatic ring, this may not influence their biological activity on *C*. *albicans* (Fig. 1). Our data demonstrate that both magnolol and honokiol have activity against the clinically isolated *C*. *albicans* strains in the concentration range of 16–32 μg/mL. According to the definition of fungistatic versus fungicidal effect [24,25], in our study magnolol and honokiol were considered to have a fungicidal effect, because cultures treated with magnolol or honokiol at MICs were unable to be recovered. It is reported that *C*. *albicans* at high cell density is tolerant to various antifungal drugs at high concentrations [33]. The results presented in this paper suggest the same behavior in the effect of magnolol and honokiol on *C*. *albicans*.

We discovered that both magnolol and honokiol at a low concentration of 4μg/mL had the potential to inhibit the transition of *C*. *albicans* from yeast to hyphae on solid spider agar plates (Fig. 3). Then, we found that the inhibitory effects of magnolol and honokiol on transition of *C*. *albicans* from yeast to hyphae were dependent on different environmental stimuli. The nutrient-poor spider medium, liquid RPMI 1640, GlcNAc, and mammalian serum are all able to induce hyphae growth [7]. Our data demonstrated that 4 μg/mL of magnolol and honokiol inhibited the hyphae growth in nutrient-poor spider medium, liquid RPMI 1640, and GlcNAc medium (Fig. 3). In contrast, 4 μg/mL of magnolol or honokiol did not suppress the hyphae growth in 10% FBS YPD medium (Fig. 3). These results suggest that environmental stimuli may need to be considered in the potential use of magnolol or honokiol as inhibitors of hyphae growth of *C*. *albicans*.

Our results further suggest that magnolol and honokiol inhibit both the formation and development of biofilm by *C*. *albicans*. Notably, magnolol and honokiol could not only inhibit the formation of biofilm but also destroy the maintenance of mature biofilm. Magnolol and honokiol at the concentration of 32 μg/mL (1×MIC) inhibited more than 90% biofilm formation in the early phase and the developmental phase. In addition, 64 μg/mL (2×MIC) of magnolol and honokiol destroyed the maintenance of about 50% mature biofilm (Fig. 4). The effects of these two compounds were similar with amphotericin B. Moreover, both compounds at a concentration of 16 μg/mL effectively reduced the thickness and viability of biofilm (Fig. 5). Furthermore, our results demonstrated that both compounds at concentrations of 8–32 μg/mL inhibited *C*. *albicans* cells adhesion to HSC-T6 cells and biofilm formation on titanium sheets (Fig. 6 and Fig. 7). In summary, our data indicate that magnolol and honokiol could inhibit biofilm formation through decreasing adhesion, morphological transition, and their fungicidal effect.

**Fig 10:**
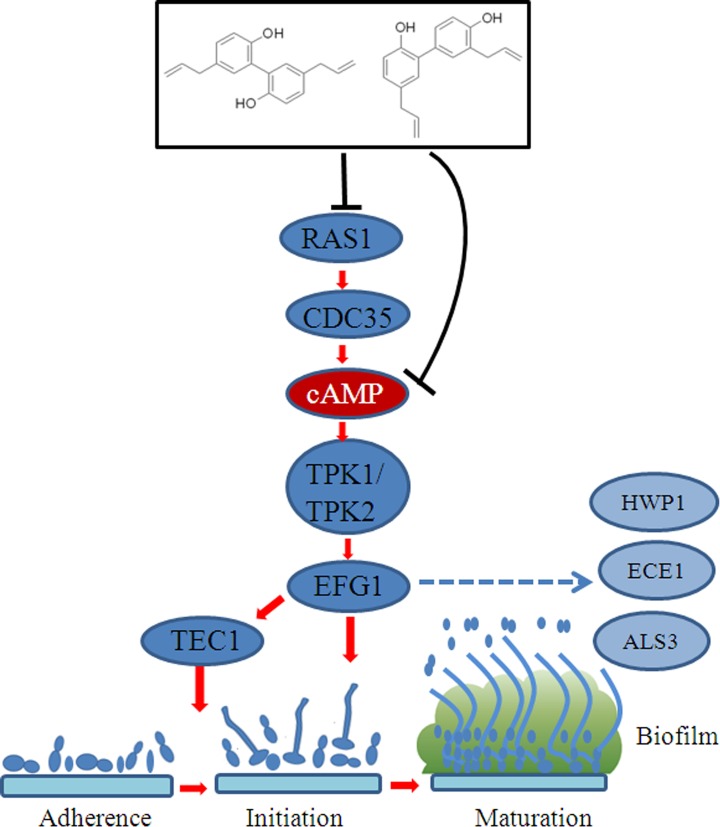
Proposed model of mechanism underlying the magnolol and honokiol-induced inhibition of biofilm formation by *C*. *albicans*. Magnolol or honokiol significantly decreased the expression levels of *RAS1*, *EFG1*, *TEC1*, and *CDC35* genes encoding the Ras1-cAMP-Efg1 pathway. In addition, exogenous cAMP restored hyphal formation in the magnolol and honokiol treatment groups. Collectively, these magnolol or honokiol-mediated effects impede the activation of the Ras1-cAMP signaling pathway and ultimately down regulate *TEC1* and *EFG1* expression thereby inhibiting hyphal growth and biofim formation. *HWP1*, *ECE1*, and *ALS3* genes which are involved in adherence are also regulated by Efg1.

Treatment with magnolol and honokiol significantly decreased expression levels of *RAS1*, *EFG1*, *TEC1*, and *CDC35*, which are members of the Ras1-cAMP-Efg1 pathway (Fig. 8A). *RAS1* encoding the Ras-family GTPase is involved in the induction of hyphal formation by activation of Ras1-cAMP-Efg1 and MAPK pathways [34]. *EFG1* and *TEC1* genes encoding transcription factors positively regulate the expression of hypha-specific genes such as *ECE1* [6],[34]. In addition, our results indicate that 3 adhesion-related genes *HWP1*, *ALS3*, and *ECE1* were significantly down-regulated following treatment with magnolol and honokiol (Fig. 8A). In *C*. *albicans*, *HWP1* which encodes a cell-wall protein is a unique adhesion gene expressed on hyphal surface. The biofilm lacking *HWP1* gene was prone to detachment from the abiotic substrate [35]. The *ALS3* gene encoding a protein similar to alpha-agglutinin plays an essential role in the adhesion of *C*. *albicans*, and serves as an upstream regulator of both the Ras1-cAMP-Efg1 and MAPK pathways [36]. *ECE1* is associated with both cell adhesion and hyphae formation by its regulation of the extent of cell elongation [37]. Interestingly, these genes: *HWP1*, *ALS3*, and *ECE1* which were down regulated after magnolol or honokiol treatment, are regulated by Ras1-cAMP-Efg1 pathway [38]. Exogenous cAMP restored hyphal formation in the magnolol and honokiol treatment groups (Fig. 8B). These results indicate that magnolol and honokiol may inhibit adhesion, transition from yeast to hyphae, and biofilm formation by *C*. *albicans* by down-regulating the Ras1-cAMP-Efg1 pathway (Fig. 10).

Using the *C*. *elegans* infection model, we found that treatment with 16 μg/mL of magnolol or honokiol increased the lifespan and reduced the CFU content of nematodes infected by *C*. *albicans* SC5314 (Fig. 9). These data further demonstrate the potential of magnolol and honokiol to be used against *C*. *albicans* infection *in vivo*.

## Conclusions

Taken together, our results suggest that magnolol and honokiol suppressed adhesion, transition from yeast to hyphae, and inhibited *C*. *albicans* biofilm formation. The molecular mechanisms of these actions may be related to the Ras1-cAMP-Efg1 pathway. We believe our study will be helpful in improving the understanding of the possible mechanisms of action of magnolol and honokiol against adhesion, transition from yeast to hyphae, and biofilm formation of *C*. *albicans*.

## Supporting Information

S1 Fig Magnolol and honokiol inhibited the adhesion of *C*. *albicans* CASA1 to HSC-T6 cells. Representative DIC and GFP fluorescent images were displayed. The treatment concentration for magnolol and honokiol was 16 μg/mL.
(TIF)

S2 Fig Expression patterns of genes encoding Ras1-cAMP-Efg1 pathway required for hyphal development and cell adhesion related genes. *C*. *albicans* YEM30 was treated with 16 μg/mL of magnolol or honokiol for 12 h at 37°C. Gene expression was indicated as a fold change relative to that of the control group after real-time RT-PCR assay. Bars represent means ± S.E.M. **P < 0.01.
(TIF)

S3 Fig Cell viability assay of HSC-T6 cells treated with different concentrations of magnolol or honokiol. Bars represent means ± S.E.M.
(TIF)

S1 Table Gene-specific primers used for real-time RT-PCR. (DOCX)

S2 Table MICs and MFCs of magnolol and honokiol for *C*. *albicans* strains used in this study. (DOC)
