# Supplementary figures and images for: Effects of Magnolol and Honokiol on Adhesion, Yeast-Hyphal Transition, and Formation of Biofilm by Candida albicans
Source: PLoS One. 2015 Feb 24;10(2):e0117695. doi: 10.1371/journal.pone.0117695 (PMC4339376; doi:10.1371/journal.pone.0117695)

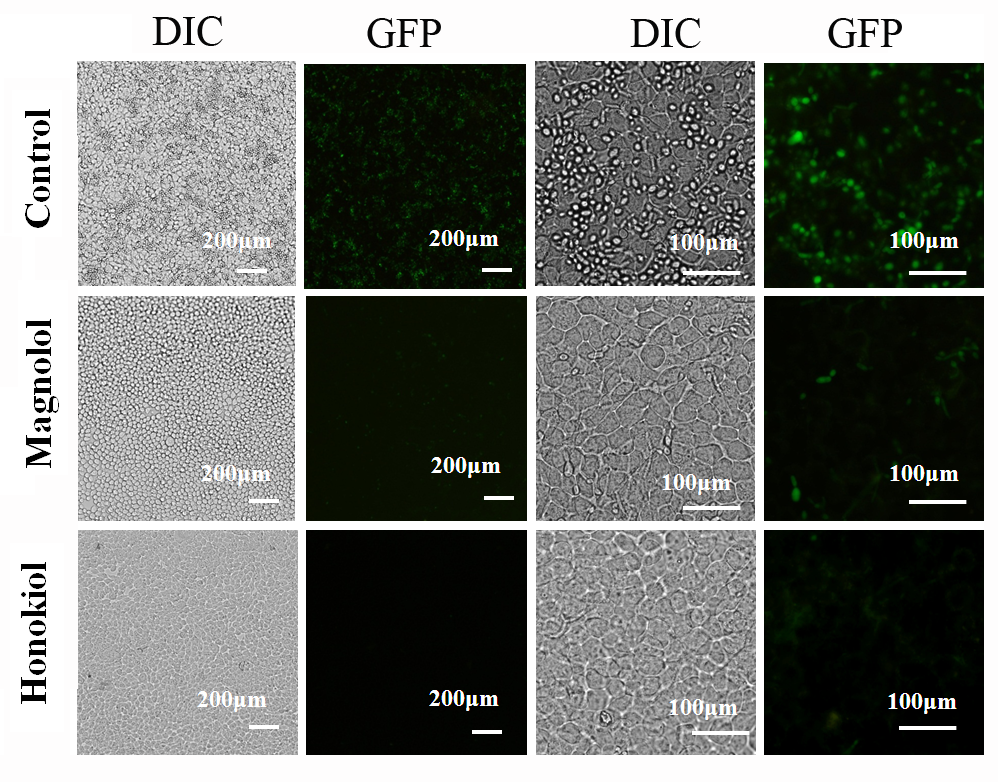

Supplement: S1 Fig — Representative DIC and GFP fluorescent images were displayed. The treatment concentration for magnolol and honokiol was 16 μg/mL. (TIF) [file pone.0117695.s001.tif]

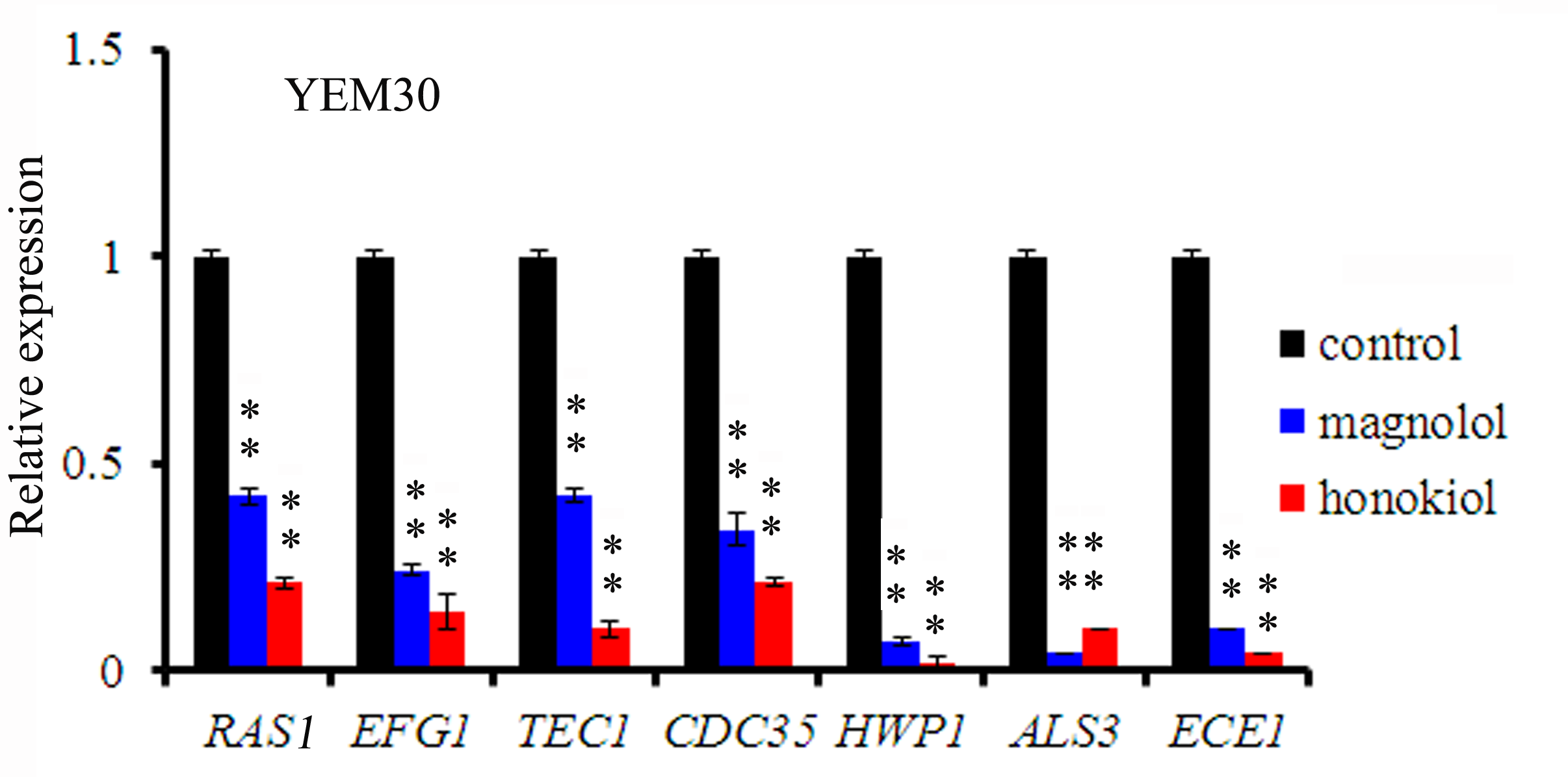

Supplement: S2 Fig — C. albicans YEM30 was treated with 16 μg/mL of magnolol or honokiol for 12 h at 37°C. Gene expression was indicated as a fold change relative to that of the control group after real-time RT-PCR assay. Bars represent means ± S.E.M. **P < 0.01. (TIF) [file pone.0117695.s002.tif]

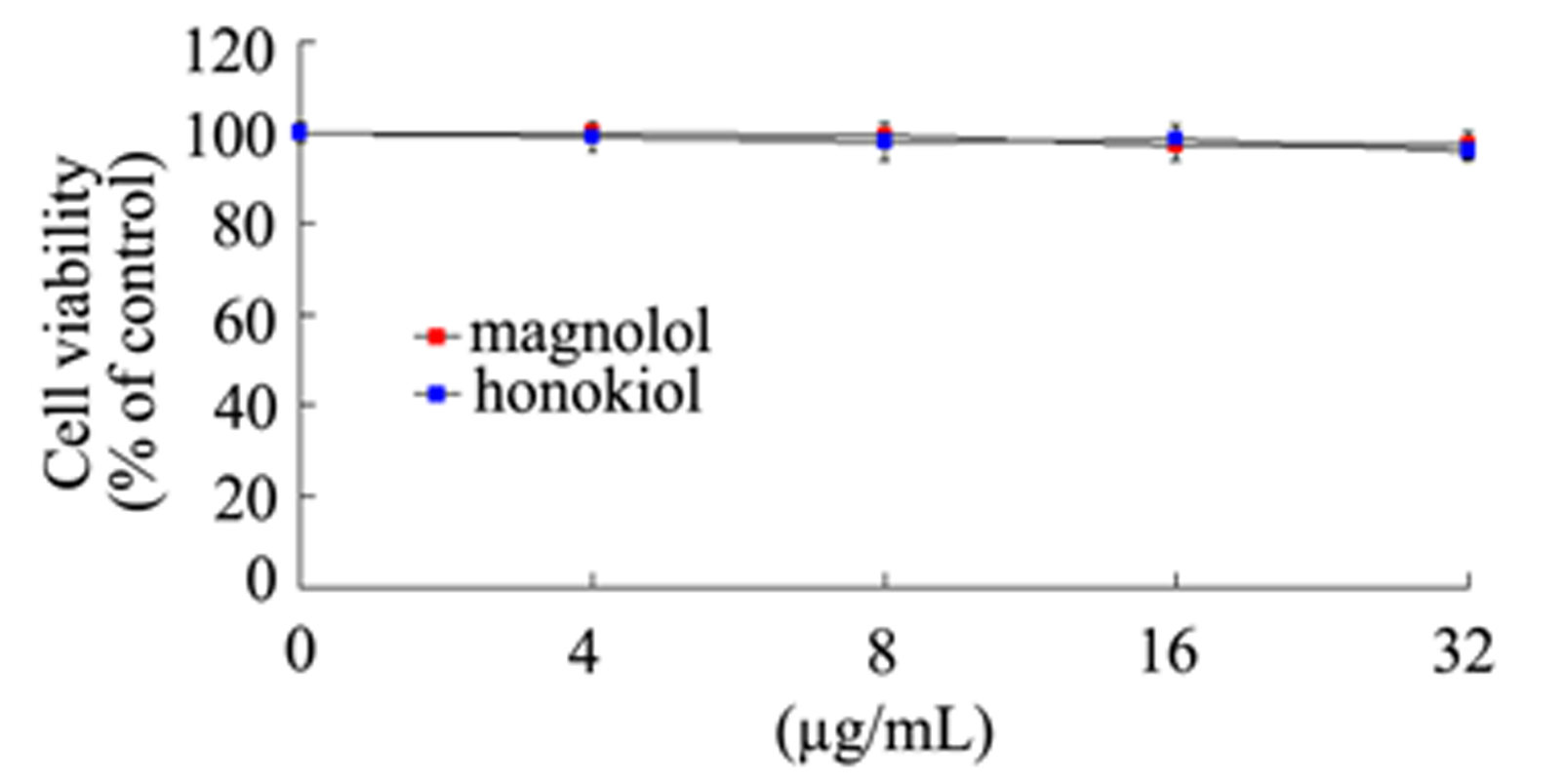

Supplement: S3 Fig — Bars represent means ± S.E.M. (TIF) [file pone.0117695.s003.tif]
