# Supplementary material for: Effects of Magnolol and Honokiol on Adhesion, Yeast-Hyphal Transition, and Formation of Biofilm by Candida albicans
Source: PLoS One. 2015 Feb 24;10(2):e0117695. doi: 10.1371/journal.pone.0117695 (PMC4339376; doi:10.1371/journal.pone.0117695)
Supplement: S1 Table — (DOCX) [file pone.0117695.s004.docx]

**Table S1. Gene-specific primers used for real-time RT-PCR**

| Gene | Forward primer (5’→3’) | Reverse primer(5’→3’) |
| --- | --- | --- |
| *GSP1* | TGAAGTCCATCCATTAGGAT | ATCTCTATGCCAGTTTGGAA |
| *RAS1* | GGCCATGAGAGAACAATATA | GTCTTTCCATTTCTAAATCAC |
| *EFG1* | TATGCCCCAGCAAACAACTG | TTGTTGTCCTGCTGTCTGTC |
| *TEC1* | AGGTTCCCTGGTTTAAGTG | ACTGGTATGTGTGGGTGAT |
| *CST20* | TTCTGACTTCAAAGACATCAT | AATGTCTATTTCTGGTGGTG |
| *HST7* | ACTCCAACATCCAATATAACA | TTGATTGACGTTCAATGAAGA |
| *CPH1* | ATGCAACACTATTTATACCTC | CGGATATTGTTGATGATGATA |
| *ALS3* | CTAATGCTGCTACGTATAATT | CCTGAAATTGACATGTAGCA |
| *HWP1* | TGGTGCTATTACTATTCCGG | CAATAATAGCAGCACCGAAG |
| *ECE1* | GCTGGTATCATTGCTGATAT | TTCGATGGATTGTTGAACAC |
| *CDC35* | TTCATCAGGGGTTATTTCAC | CTCTATCAACCCGCCATTTC |
