# Supplementary material for: Effects of Magnolol and Honokiol on Adhesion, Yeast-Hyphal Transition, and Formation of Biofilm by Candida albicans
Source: PLoS One. 2015 Feb 24;10(2):e0117695. doi: 10.1371/journal.pone.0117695 (PMC4339376; doi:10.1371/journal.pone.0117695)
Supplement: S2 Table — (DOC) [file pone.0117695.s005.doc]

**Table S2. MICs and MFCs of magnolol and honokiol for *C. albicans* strains used in this study.**

| Strains | MIC (µg/mL) | |  | MFC (µg/mL) | |
| --- | --- | --- | --- | --- | --- |
|  | Magnolol | Honokiol |  | Magnolol | Honokiol |
| SC5314 | 32 | 32 |  | 32 | 32 |
| CA1 | 16 | 16 |  | 16 | 16 |
| CA2 | 16 | 16 |  | 16 | 16 |
| CA3 | 16 | 16 |  | 16 | 16 |
| CA4 | 16 | 16 |  | 16 | 16 |
| CA10 | 32 | 32 |  | 32 | 32 |
| CA127 | 16 | 16 |  | 16 | 16 |
| CA129 | 16 | 16 |  | 16 | 16 |
| CA132 | 16 | 16 |  | 16 | 16 |
| CA135 | 32 | 32 |  | 32 | 32 |
| CA137 | 32 | 32 |  | 32 | 32 |
| CASA1 | 32 | 32 |  | 32 | 32 |
| YEM30 | 32 | 32 |  | 32 | 32 |
